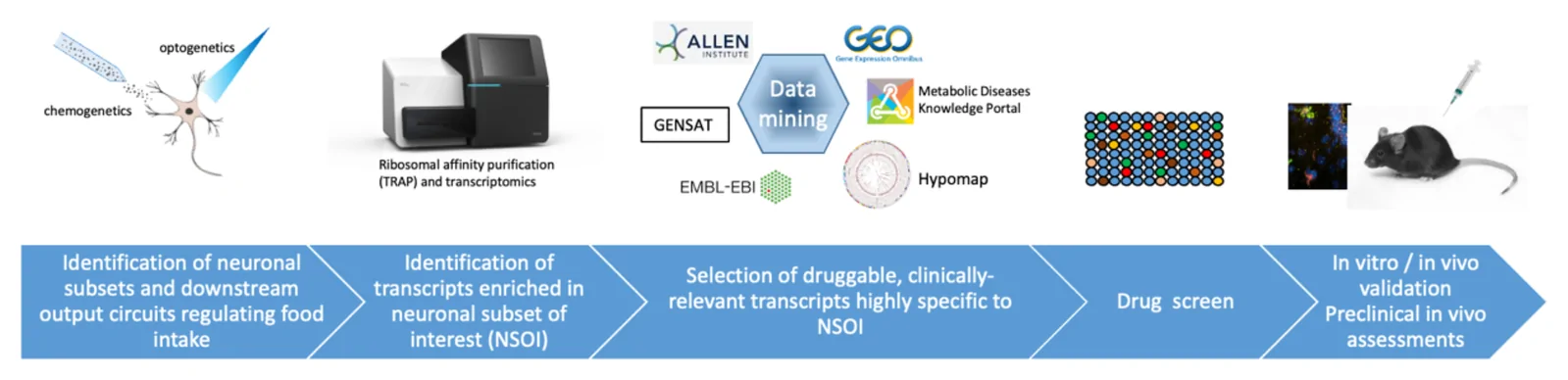# A pipeline for identification and validation of brain targets for weight loss

**DOI:** 10.1038/s41574-023-00803-w

**Published:** 2023-04-01

**Authors:** Anthony Tsang, Clemence Blouet

**Affiliations:** 1 Wellcome-MRC Institute of Metabolic Science and Medical Research Council Metabolic Disease Unit, University of Cambridge; Cambridge, UK

Obesity, a major risk factor for cardiometabolic diseases and various forms of cancer, represents one of the greatest health challenges of the modern world. While the relative contributions of genetic, socioeconomic and dietary factors remain controversial, recent progress in our understanding of the biological pathways controlling appetite and energy balance offer new prospects of curbing the obesity epidemic. Unlike the first generation of anti-obesity drugs that failed to combine safety and efficacy, a new class of anti-obesity agents leveraging the appetite-suppressing effect of GLP-1R agonism has emerged, producing spectacular weight loss profiles in obese patients. This has catalysed a renewed interest in the discovery of anti-obesity drugs and highlighted the need to identify drug combinations to further increase weight loss responses to GLP-1 receptor agonists, alternative drugs treating forms of obesity that do not respond to incretin-based therapies, and strategies for long-term weight loss maintenance.

In an article published in Nature Metabolism, Schneeberger et al. [1] identify an anti-obesity drug target in the dorsal raphe nucleus (DRN), a brain area in the dorsomedial midbrain below the cerebral aqueduct. The DRN is molecularly diverse and extensively connected, and controls various functions including food intake and energy expenditure. Following up on the identification of appetite-suppressing glutamatergic neurons of the dorsal raphe nucleus (DRN^Vglut3^) [2], the authors test a pipeline for anti-obesity drug target identification and validation using transcriptomics analysis, druggable target selection based on DRN^Vglut3^ transcript enrichment, and energy balance assessments following pharmacological targeting of top candidates, including Hcrtr1 (hypocretin receptor 1). Pharmacological administrations of non-selective Hctr antagonists into the DRN or whole brain suppress food intake and produce weight loss in obese mice, but also increase somnolence. They next assessed the effects of CVN45502, a highly selective Hcrtr1-antagonist with good brain penetration and pharmacokinetic properties. Oral treatment with CVN45502 reduces food intake and prevents further weight gain in obese mice without modifying sleeping patterns. The authors conclude the paper by showing co-expression of *VGLUT3* and *HCRTR1* in the human DRN, laying foundations for clinical trials testing the weight loss action of CVN45502 in obese patients.

Although the DRN has received less attention in metabolic research than other brainstem nuclei such as the nucleus of the solitary tract or parabrachial nucleus, it is nonetheless one of the key serotonergic nuclei of the mammalian brain. Given the history of pharmacotherapies targeting serotonin (5HT) receptors to treat hyperphagic obesity, and the fact that DRN^Vglut3^ neurons largely co-express 5HT [2], it would be interesting to determine the role of serotonergic transmission in the functional consequences of DRN^Vglut3^ activation. The authors provide evidence that DRN^Vglut3^ neurons do not densely innervate the arcuate nucleus of the hypothalamus (ARH), a key neuroanatomical substrate for the appetite-suppressive action of 5HT receptor agonists. Thus, *Vglut3* might specifically label a subset of DRN^5HT^ neurons that do not project to the ARH, consistent with the lack of hypothalamic projections of DRN^Vglut3/5HT^ neurons [3]. Intriguingly, *Hcrtr1* and *Hcrtr2* are expressed in largely segregated DRN^5HT^ neuronal subsets with a dominance of *Hcrtr1*, but the majority of DRN^Vglut3/5HT^ neurons express *Hcrtr2* [3, 4]. Thus, Hcrtr1^+^/5HT^+^ neurons might in fact represent only a minority of DRN^Vglut3^ neurons. In-depth mining of available DRN single-cell transcriptomic databases could provide a high-resolution description of the molecularly diversity of DRN^Vglut3^ neurons and help predict whether strategies targeting these neurons engage widespread serotonergic outputs. Excitingly, several reports indicate strong molecular, neuroanatomical and functional segregation of DRN neurons [3, 5], opening the possibility of refined targeting of DRN populations with minimal off-target effects.

A major challenge of the proposed drug discovery pipeline is the successful identification of druggable targets specifically expressed in the neuronal population of interest (here DRN^Vglut3^). Here, although *Hcrtr1* is highly enriched in the DRN^Vglut3^ neurons, it is also expressed and functionally relevant in other brain nuclei including the ventral tegmental area and locus coeruleus. Whether these additional brain targets are activated by orally-delivered CVN45502 and contribute to its appetite-suppressive action remains to be established. Furthermore, *Hcrtr1* KO mice harbour changes in anxiety, depression-like behaviour and social interactions [6], suggesting that CVN45502 treatment might alter the activity of *Hcrtr1*^*+*^ targets regulating these behaviours and produce similar phenotypes. Nevertheless, it is exciting to think that taking this work a step further, we might in the future be able to obtain projection-specific molecular footprints allowing the specific activation of molecularly- and functionally- distinct subsets of DRN neurons.

One aspect that would be interesting to eventually integrate in this drug target discovery pipeline is human genetics data. Interestingly, human genetic variants in HCRTR1 are significantly associated with BMI (https://t2d.hugeamp.org/), giving further confidence that HCRTR1 represents a relevant target to treat human obesity, but associate with naps and day-time sleepiness as well, pointing at potential species differences. The ability to add this level of information and select for functionally relevant molecular markers for neuronal subsets of interest would further expand the value of this pipeline.

One aspect that is not fully elucidated in the study from Schneeberger et al. is the nature of the behavioural effectors mediating the anorexic response to chemogenetic or pharmacological activation of DRN^Vglut3^ neurons. Photoactivation of their proposed downstream target LH (lateral hypothalamus)^Vglut2^ neurons is aversive [7], raising the possibility that the observed feeding-suppressive response are secondary to the production of visceral malaise and conditioned-taste avoidance. This might limit the possibility of combining GLP-1R agonists with CVN45502 or other drugs targeting DRN^Vglut3^ neurons. Alternatively, or in addition to this mechanism, activation of DRN^Vglut3^ neurons might modulate hedonic controls of food intake, particularly relevant in diet-induced obese mice [8].

The data presented by Schneeberger et al. delineate intriguingly complex reciprocal communications between the DRN and the LH, a canonical feeding-regulatory center. On one hand, the authors conclude that DRN^Vglut3^ neurons project to LH^Vglut2^ neurons, which suppress feeding [7] but also largely co-express orexin [9]. On the other hand, the DRN receives inputs from the LH [10] and inhibition of orexinergic signalling in the DRG suppresses feeding. How endogenous orexinergic inputs might dynamically modulate DRN^Vglut3^ neurons in physiological situations and possibly produce a negative feedback modulation of DRN^Vglut3^ neurons remains to be established.

Finally, the authors propose the attractive idea that the circuit described here lies downstream from leptin-receptor expressing neurons and represents an opportunity to bypass leptin-resistant circuits in obese patients. In support for this idea, it would be interesting to evaluate the activity of DRN neurons under various nutritional states in mice with dysfunctional leptin signalling, or test the consequences of ARH leptin signalling on DRN^Vglut3^ neuronal activation. Alternatively, this circuit might be independent of leptin signalling. In fact, DRN neurons respond to a variety of interoceptive cues and DRN^Vglut3^ neurons were primarily identified for their high activation pattern following a refeed [2]. DRN neurons also receive inputs from various medullary regions including the NTS, and might relay gut-related inputs to the forebrain [10].

Collectively, this study highlights the value of the combination of modern neuroscience techniques, transcriptomics technologies, open -omics databases and high-throughput drug screens for the identification of weight-loss agents, giving exciting new perspectives for future anti-obesity drug discovery (Figure 1).

## Figures and Tables

**Figure:**